# Supplementary material for: Long-lived efficient delayed fluorescence organic light-emitting diodes using n-type hosts
Source: Nat Commun. 2017 Dec 21;8:2250. doi: 10.1038/s41467-017-02419-x (PMC5740151; doi:10.1038/s41467-017-02419-x)
Supplement: Supplementary file 1 — Supplementary Information [file 41467_2017_2419_MOESM1_ESM.pdf]

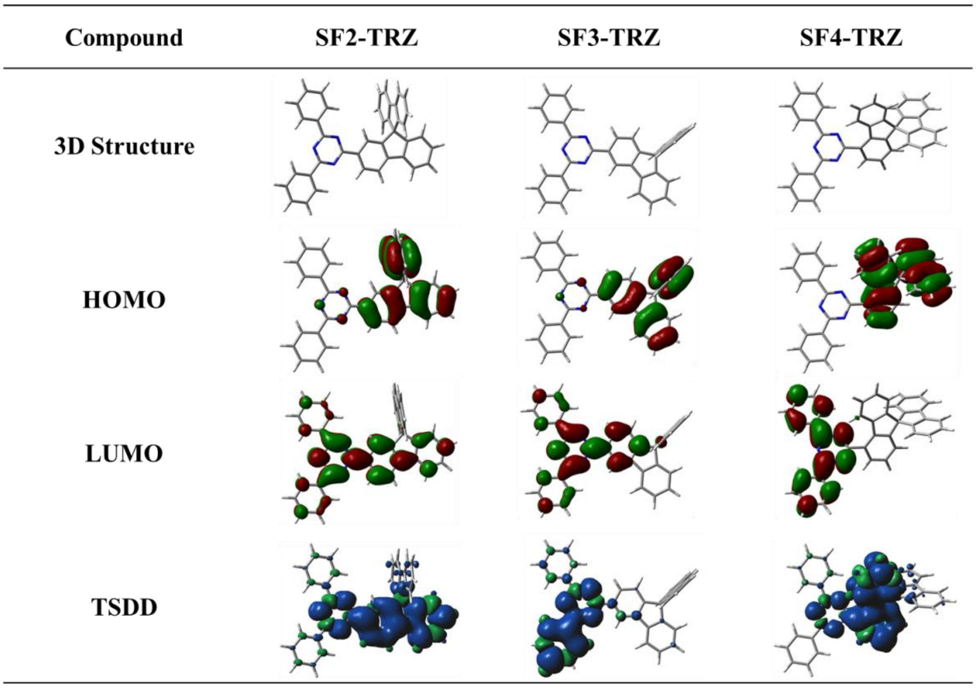

Supplementary Fig. 1 | Calculated spatial distributions of HOMO, LUMO and triplet spin density (TSDD) of SF2-TRZ, SF3-TRZ, and SF4-TRZ.

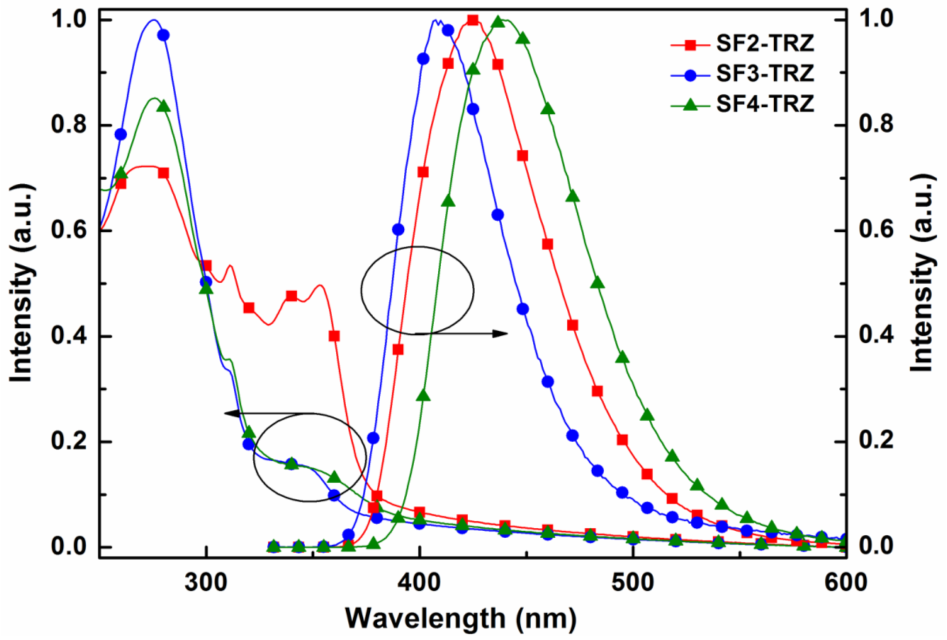

Supplementary Fig. 2 | UV-Vis absorption spectra and PL emission spectra of SF2-TRZ, SF3-TRZ, and SF4-TRZ in neat film states.

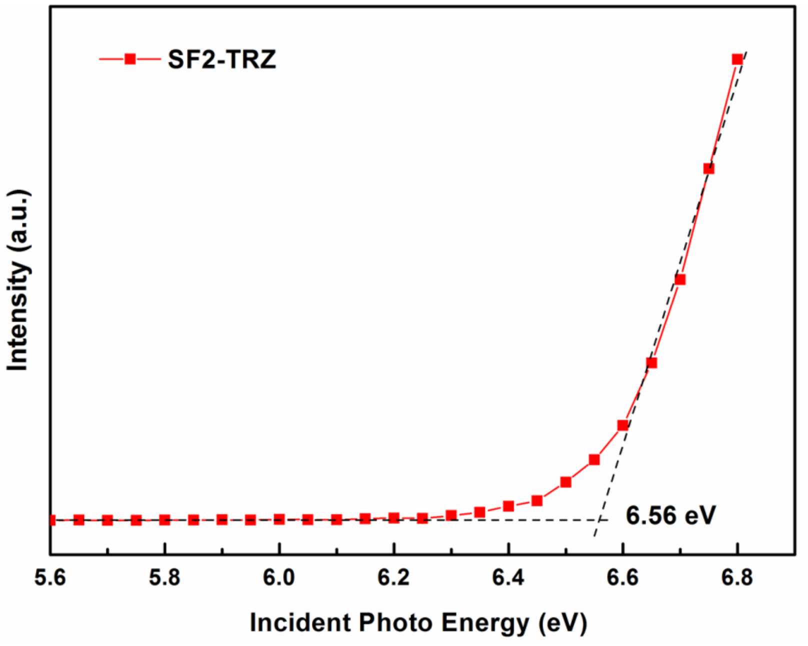

Supplementary Fig. 3 | Photoelectron spectral measurement of SF2-TRZ neat film under nitrogen atmosphere.

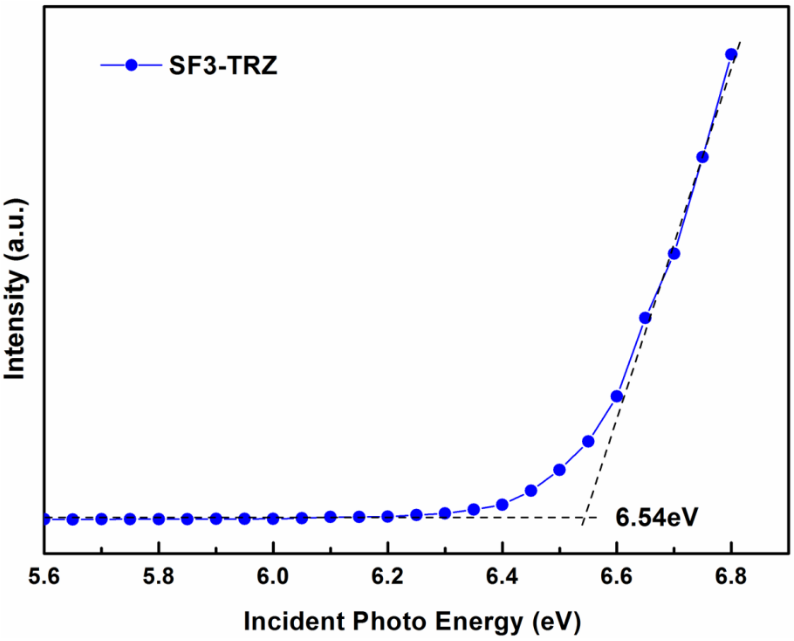

Supplementary Fig. 4 | Photoelectron spectral measurement of SF3-TRZ neat film under nitrogen atmosphere.

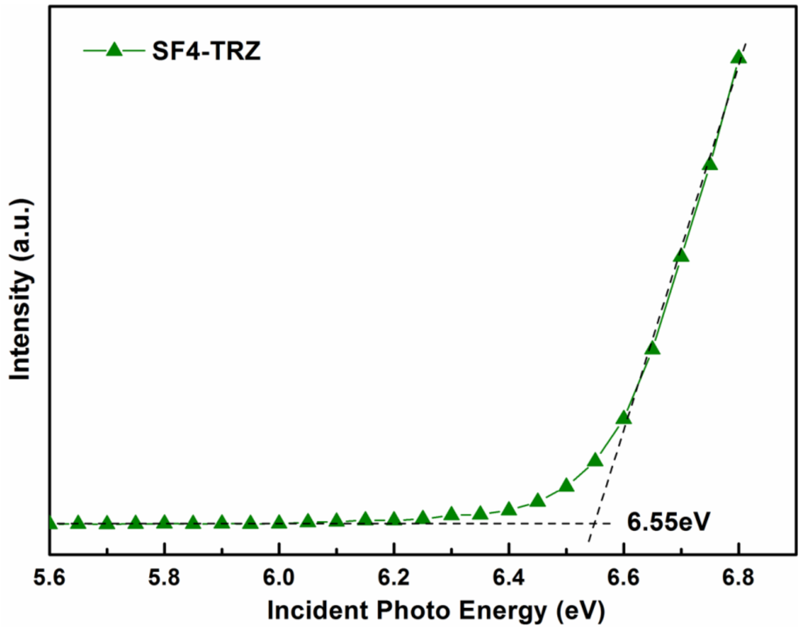

Supplementary Fig. 5 | Photoelectron spectral measurement of SF4-TRZ neat film under nitrogen atmosphere.

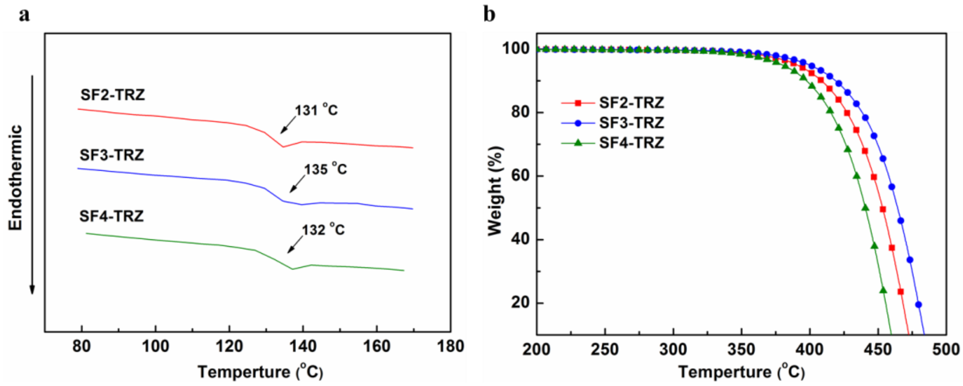

Supplementary Fig. 6 | Thermal stabilities of compounds SF2-TRZ, SF3-TRZ and SF4-TRZ. **a,** TGA thermograms of compounds SF2-TRZ, SF3-TRZ and SF4-TRZ. The temperatures shown in the figure correspond to the 5% weight loss. **b,** DSC thermograms of SF2-TRZ, SF3-TRZ and SF4-TRZ.

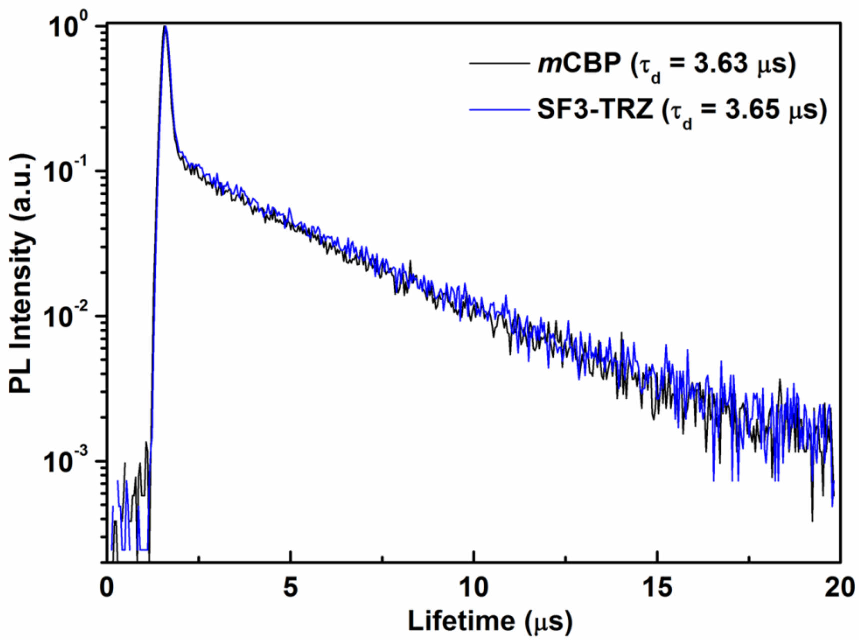

Supplementary Fig. 7 | Transient PL decays of *m*CBP and SF3-TRZ films doped with 15wt % 4CzIPN.

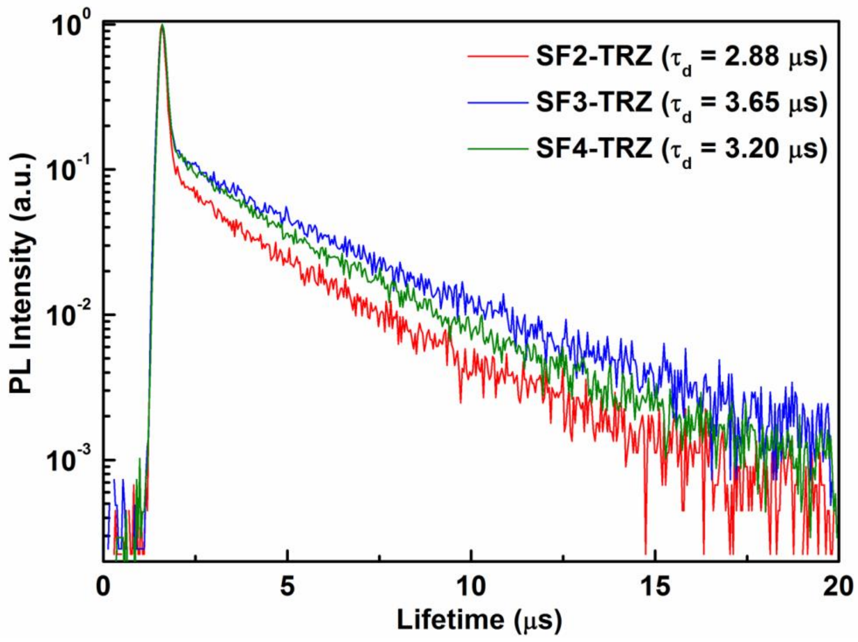

Supplementary Fig. 8 | Transient PL decays of SF2-TRZ, SF3-TRZ and SF4-TRZ films doped with 15wt % 4CzIPN.

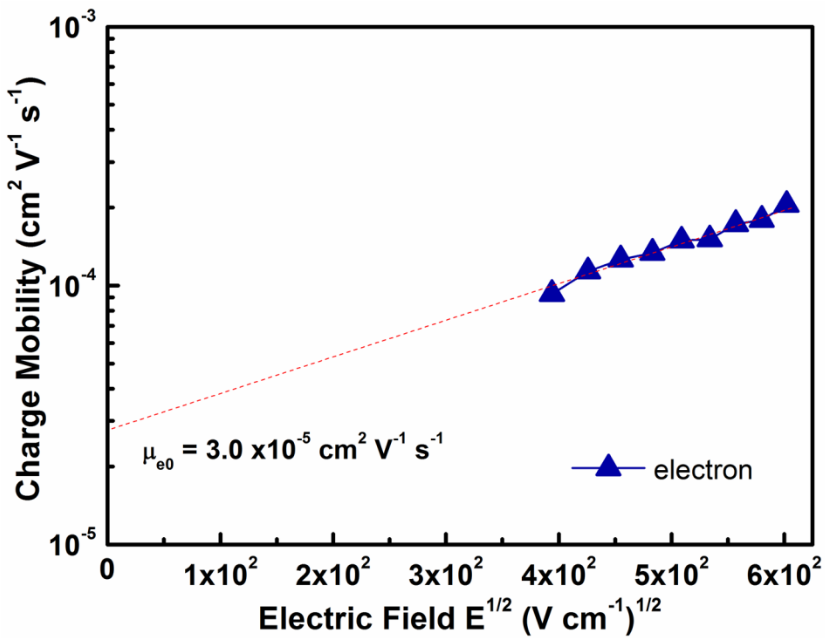

Supplementary Fig. 9 | Electron mobility plotted with respect to $E^{1/2}$ for SF3-TRZ.

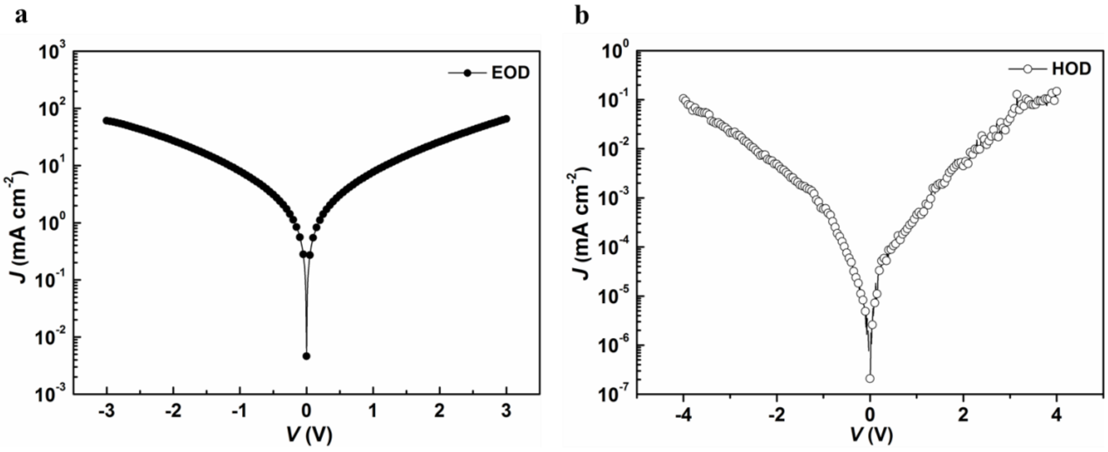

Supplementary Fig. 10 | *J*–*V* characteristics of SF3-TRZ single-carrier devices. **a,** *J*–*V* characteristics of electron-only device (ITO/Cs (10 nm)/SF3-TRZ (100 nm)/Cs (10 nm)/Al (100 nm)) in forward and reverse bias directions. **b,** *J*–*V* characteristics of hole-only device (ITO/MoO_3 (1 nm)/SF3-TRZ (100 nm)/MoO_3 (10 nm)/Al (100 nm) in forward and reverse bias directions.

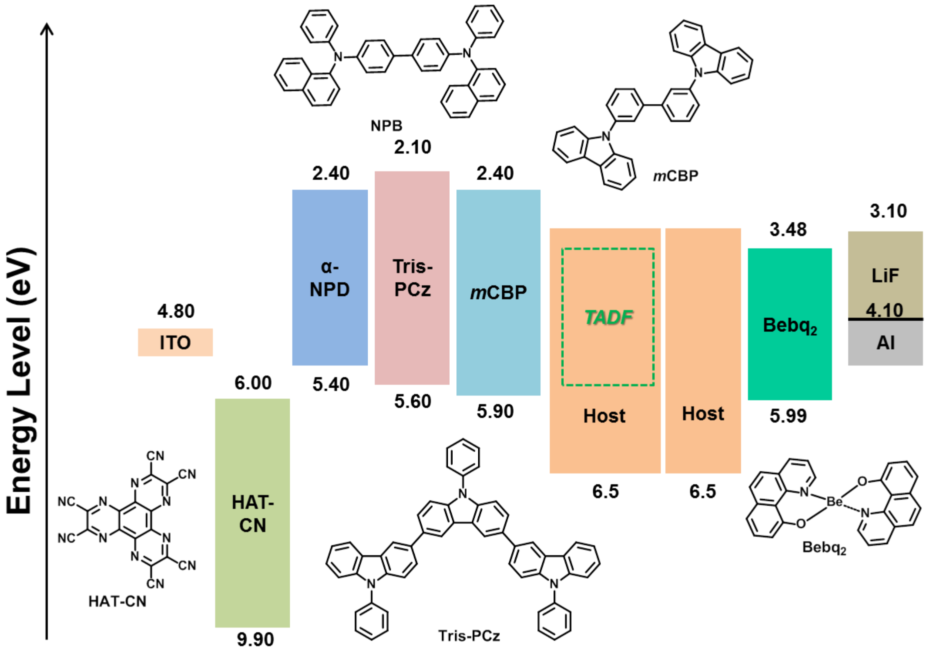

Supplementary Fig. 11 | Energy levels of the materials employed in the devices.

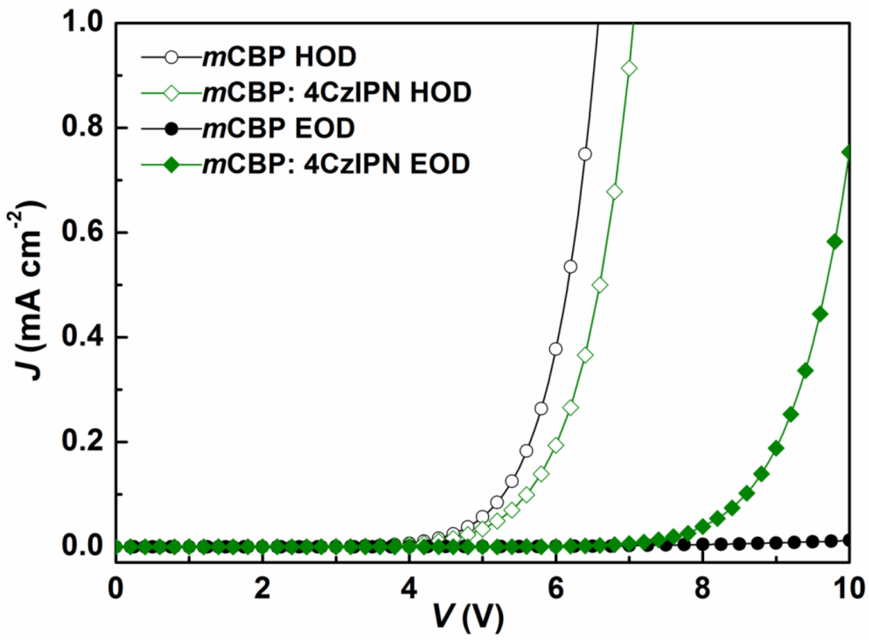

Supplementary Fig. 12 | *J*–*V* curves for the hole-only devices (ITO/ HAT-CN (10 nm)/ α-NPD (30 nm)/ Tris-PCz (20 nm)/ *m*CBP (10 nm)/mCBP: *x* wt% TADF (100 nm)/ Tris-PCz (20 nm)/Al (100 nm), where *x*= 0 or 15) and the electron-only devices (ITO/T2T (20 nm)/ *m*CBP: *x* wt% TADF (100 nm)/ T2T (10 nm)/ $Bebq_2$ (35nm)/ LiF (0.8 nm)/Al (120 nm), where *x*= 0 or 15).

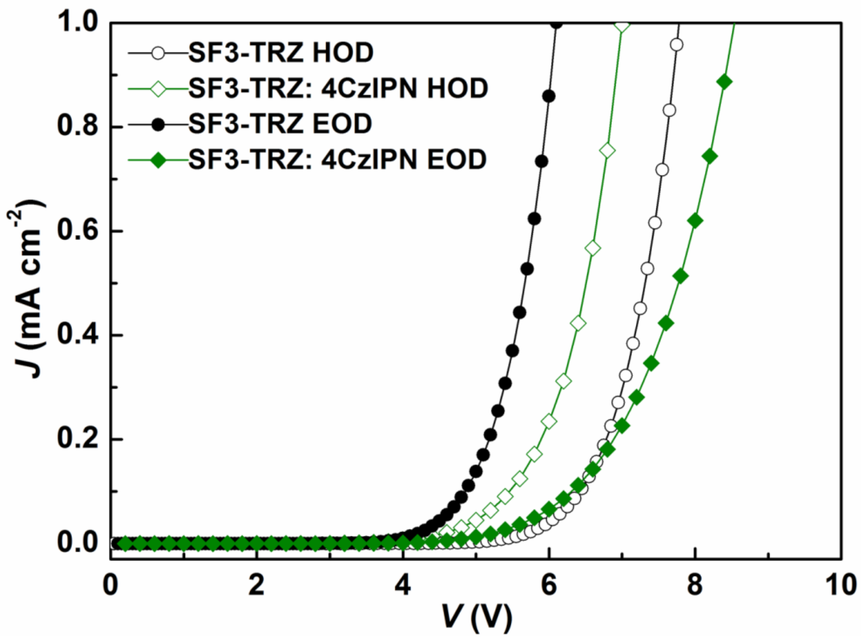

Supplementary Fig. 13 | *J*–*V* curves for the hole-only devices (ITO/ HAT-CN (10 nm)/ α-NPD (30 nm)/ Tris-PCz (20 nm)/ *m*CBP (10 nm)/SF3-TRZ: *x* wt% TADF (100 nm)/ Tris-PCz (20 nm)/Al (100 nm), where *x*= 0 or 15) and the electron-only devices (ITO/ SF3-TRZ (20 nm)/ SF3-TRZ: *x* wt% TADF (100 nm)/ SF3-TRZ (10 nm)/ $Bebq_2$ (35nm)/ LiF (0.8 nm)/Al (120 nm), where *x*= 0 or 15).

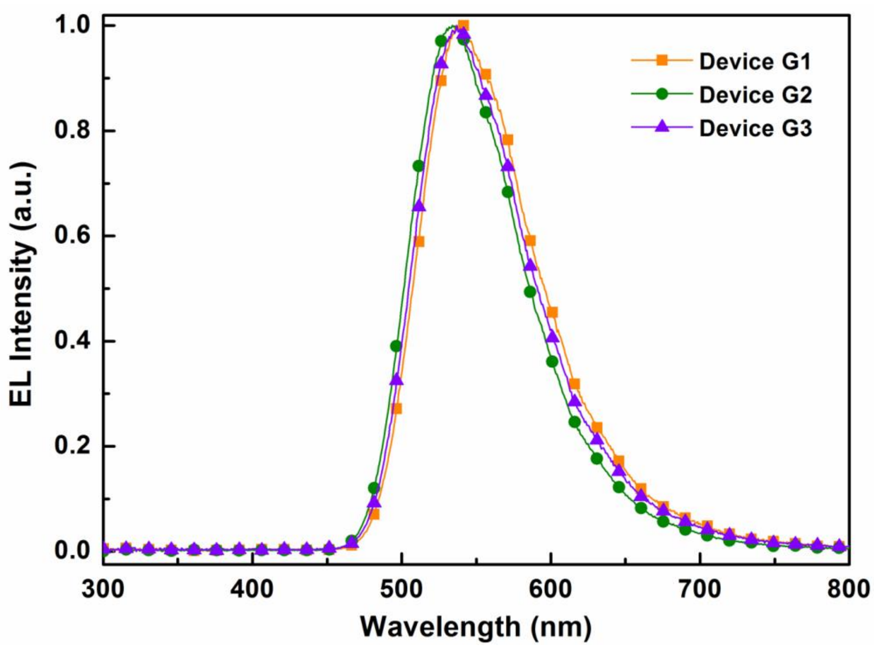

Supplementary Fig. 14 | The EL spectrum at 10 mA/cm^2 for the device G1, G2 and G3.

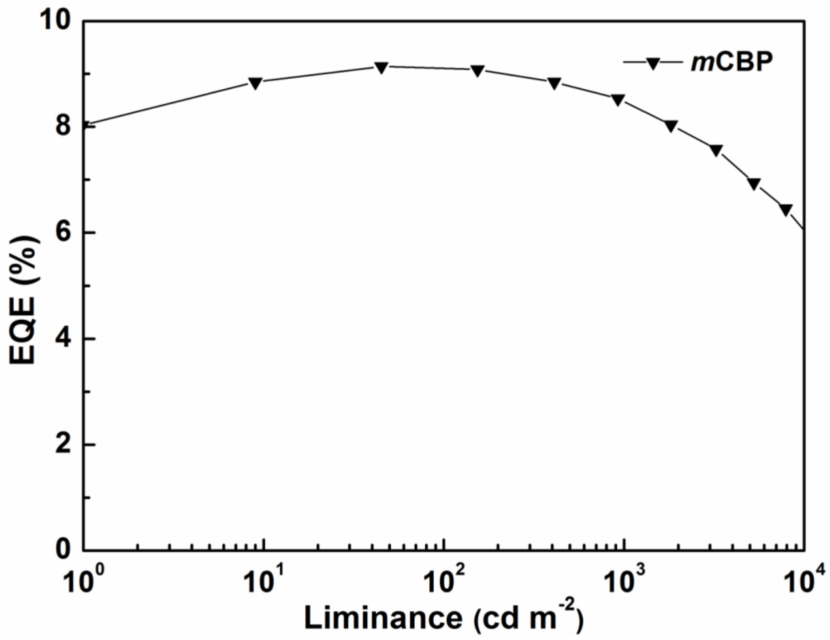

Supplementary Fig. 15 | EQE versus luminance curve of *m*CBP-based device.

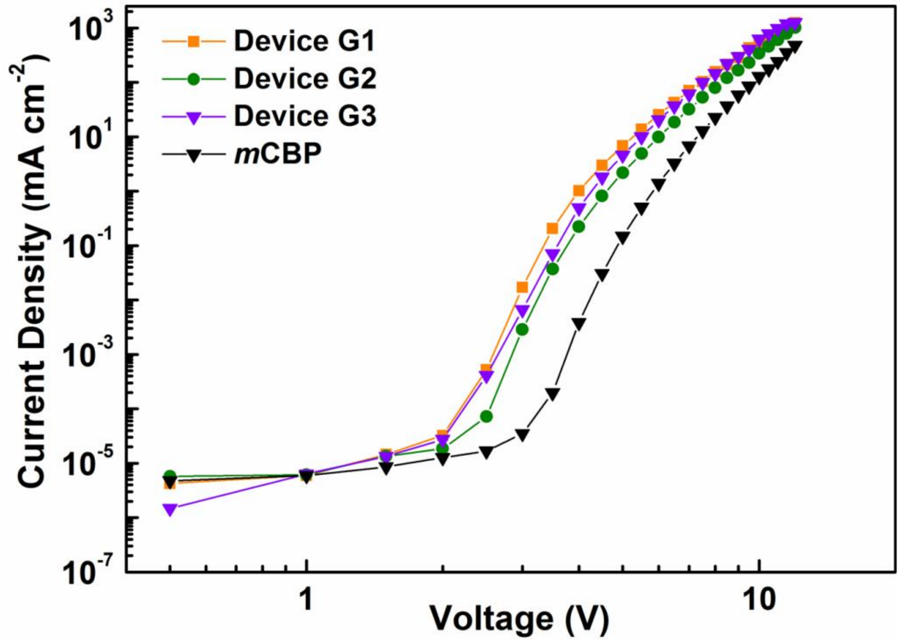

Supplementary Fig. 16 | *J*–*V* curves of device G1, G2, G3 and *m*CBP-based device.

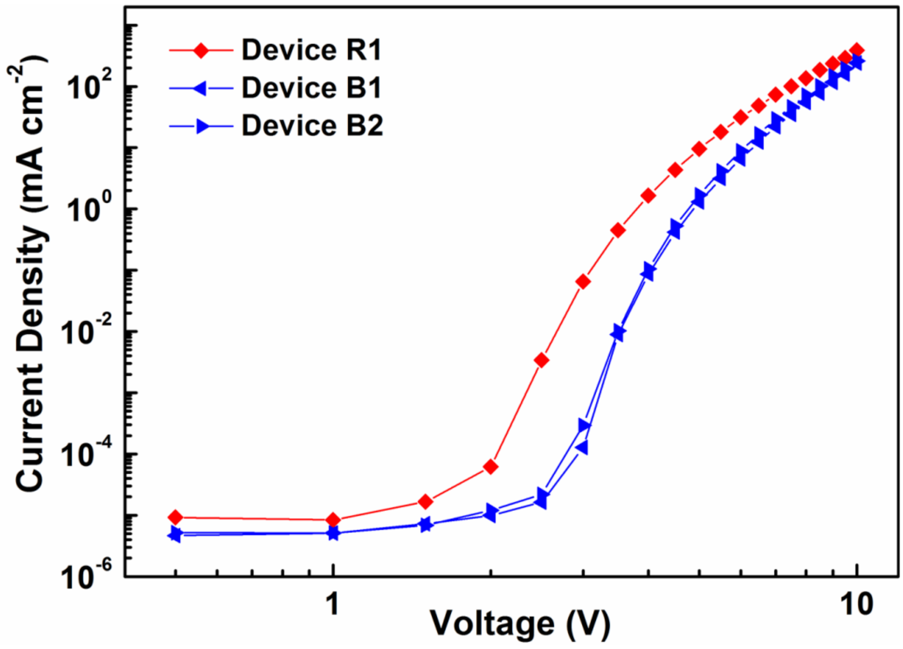

Supplementary Fig. 17 | *J–V* curves of device R1, B2 and B3.

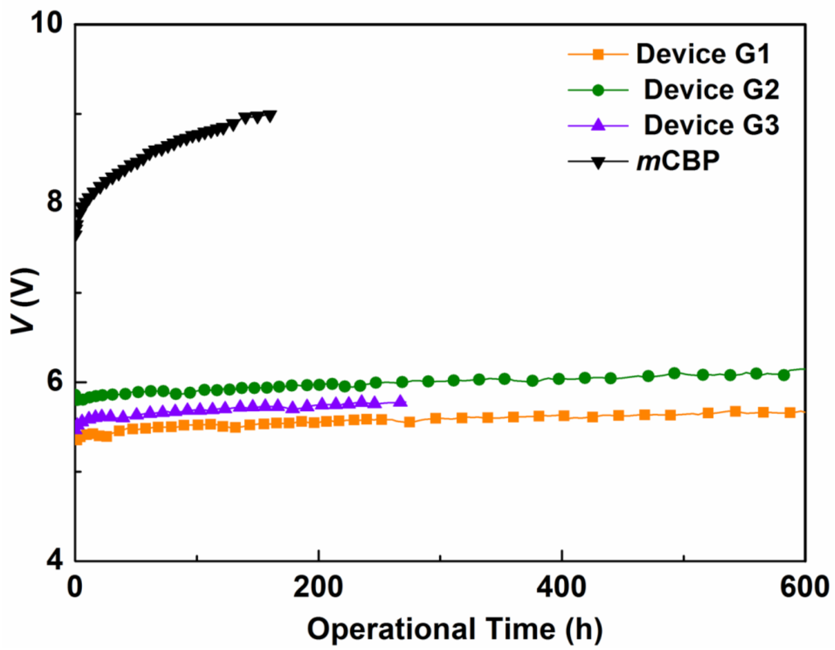

Supplementary Fig. 18 | Changes in voltage (*V*) of the devices G1, G2, G3 and *m*CBP-based device versus operational time during which the devices driven at an initial luminescence of 5,000 cd/cm^2.

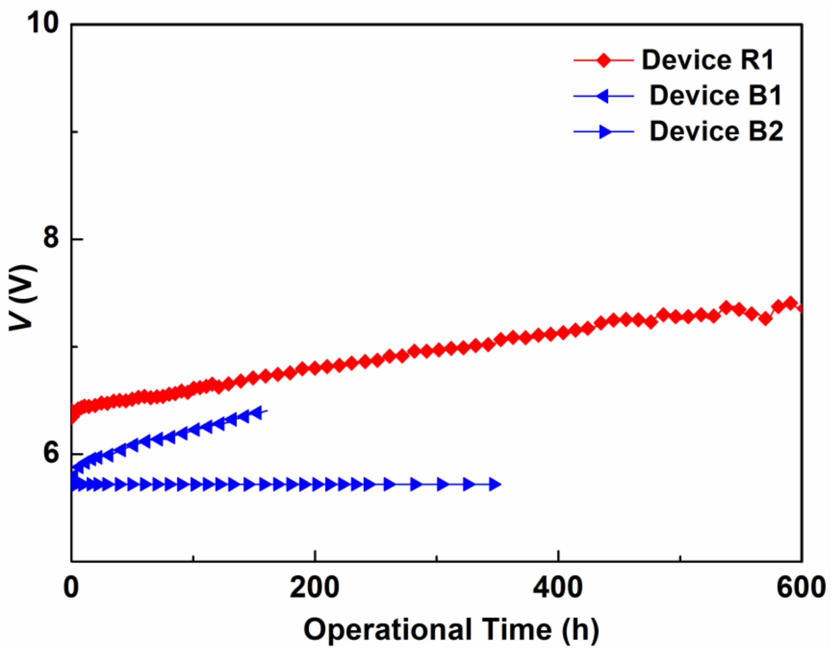

Supplementary Fig. 19 | Changes in voltage (*V*) of the devices R1, B1 and B2 versus operational time during which the devices R1, B1 and B2 driven at an initial luminescence of 2,000 cd/cm^2, 1,000 cd/cm^2 and 1,000 cd/cm^2, respectively.

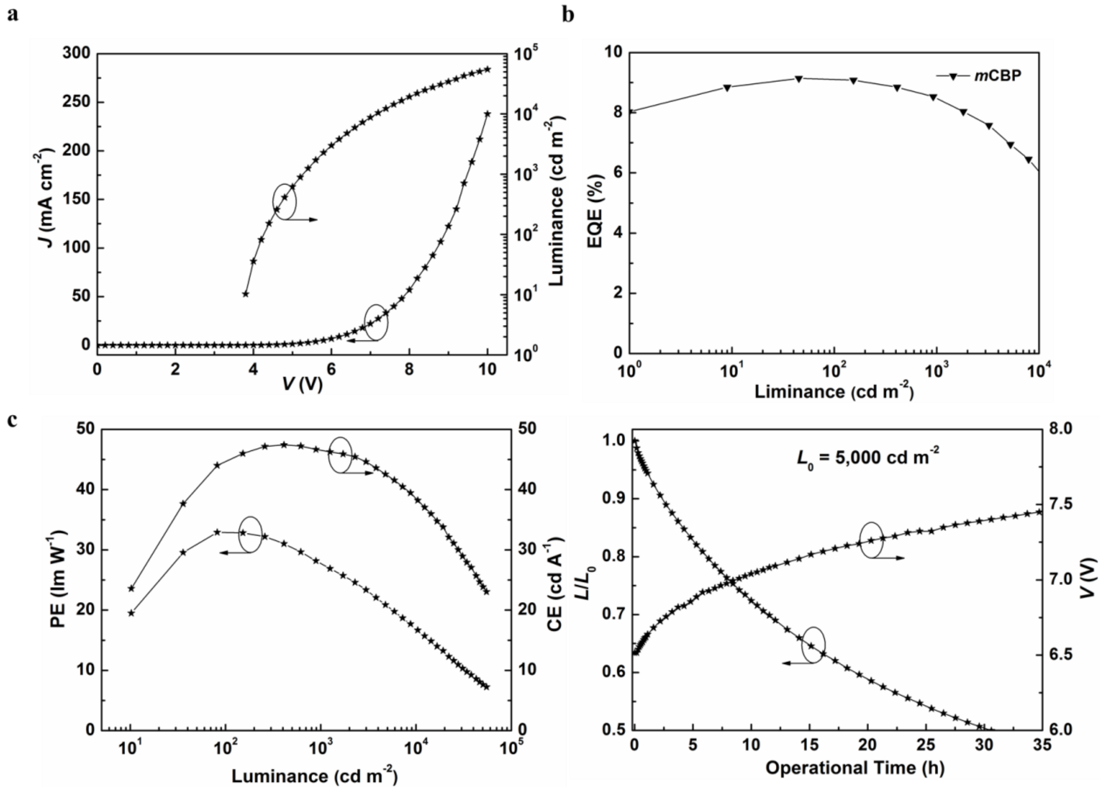

Supplementary Fig. 20 | Performance of OLED with structure ITO/HAT-CN (10 nm)/Tris-PCz (30 nm)/*m*CBP (10 nm)/mCBP: 15 wt% 4CzIPN (30 nm)/T2T (10 nm)/BPy-TP2 (35 nm)/LiF (0.8 nm)/Al (120 nm). **a,** *J*–*V*–luminance (*L*) characteristics. **b,** EQE versus luminance. **c,** Power efficiency (PE) and current efficiency (CE) versus luminance. **d,** Normalized EL intensity and voltage (*V*) rise as a function of operational time at an initial luminance of 5,000 cd/m^2. (Note that the device was fabricated under vacuum at pressures below $3.0\ 10^{-4}$ Pa and all layers (organic layers and metal electrodes) were deposited in the same evaporation chamber).

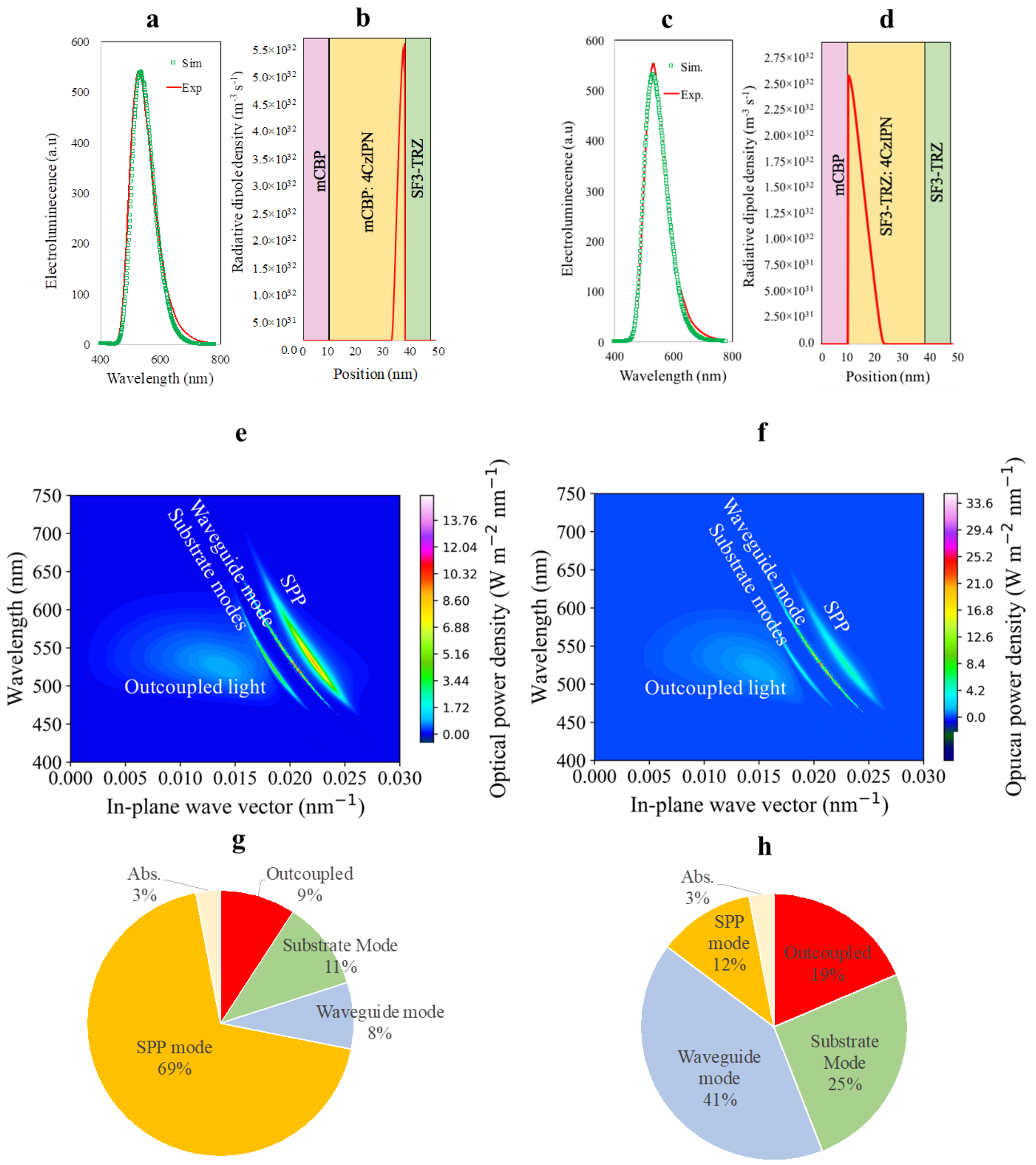

Supplementary Fig. 21 | Optical simulations of TADF OLEDs based on p-type and n-type host. **a** and **c,** Simulated and experimental EL spectra for *m*CBP-based OLED and SF3-based OLED, respectively. **b** and **d,** Recombination zone (Radiative dipole) distribution and position in *m*CBP-based OLED and SF3-based OLED, respectively. **e** and **f**, Calculated total dissipated optical power density for *m*CBP-based OLED and SF3-based OLED, respectively. **g** and **h**, Amount of the optical power coupled to the different optical channels in *m*CBP-based OLED and SF3-based OLED, respectively.

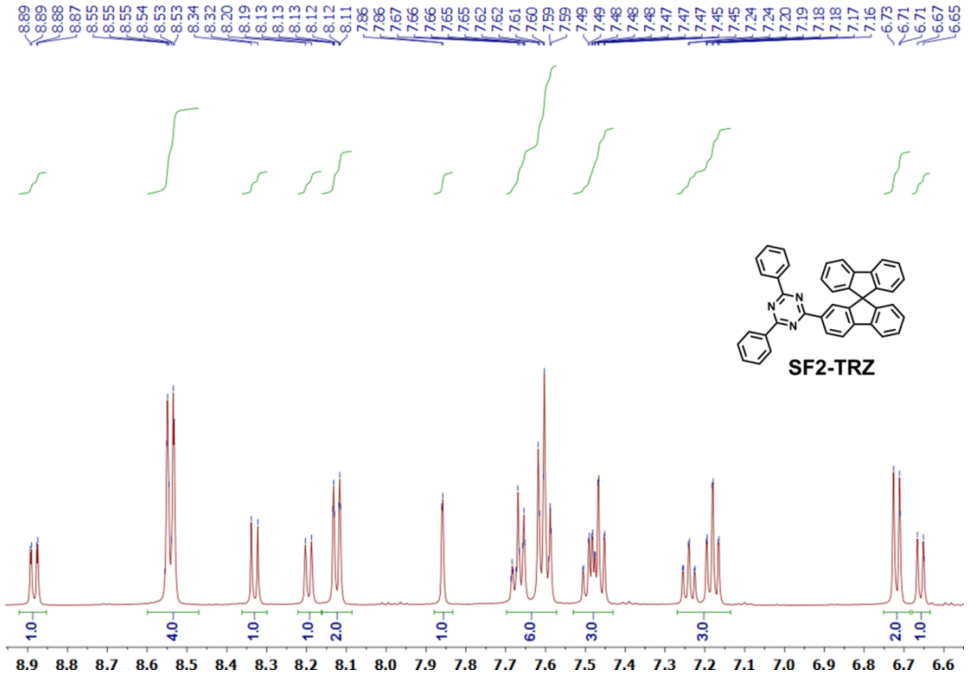

Supplementary Fig. 22 | ^{1}H NMR spectrum of SF2-TRZ.

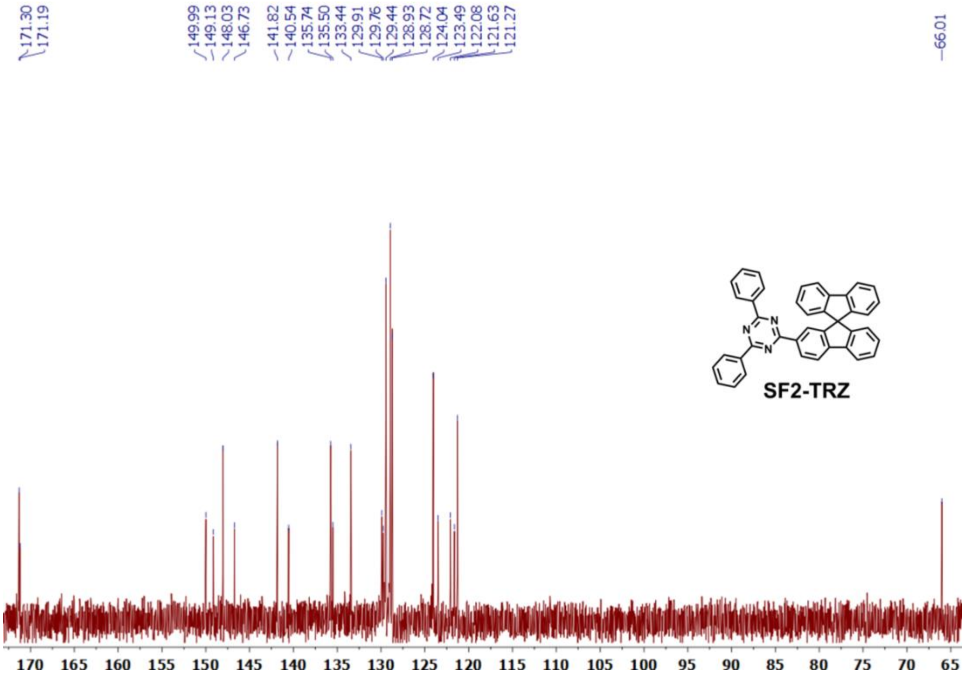

Supplementary Fig. 23 | ^{13}C NMR spectrum of SF2-TRZ.

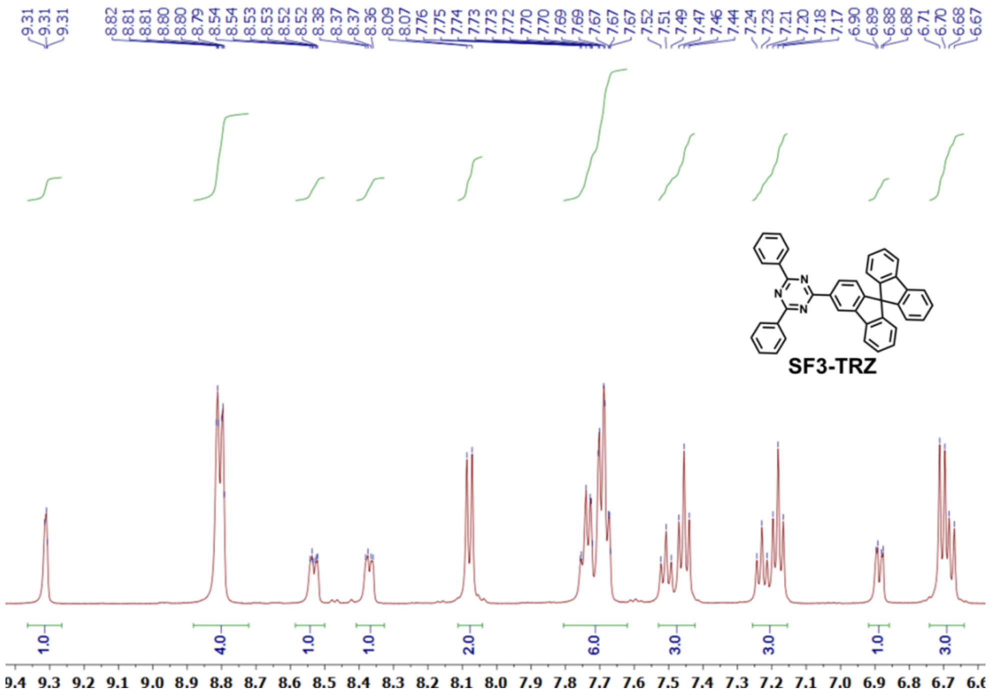

Supplementary Fig. 24 | ^{1}H NMR spectrum of SF3-TRZ.

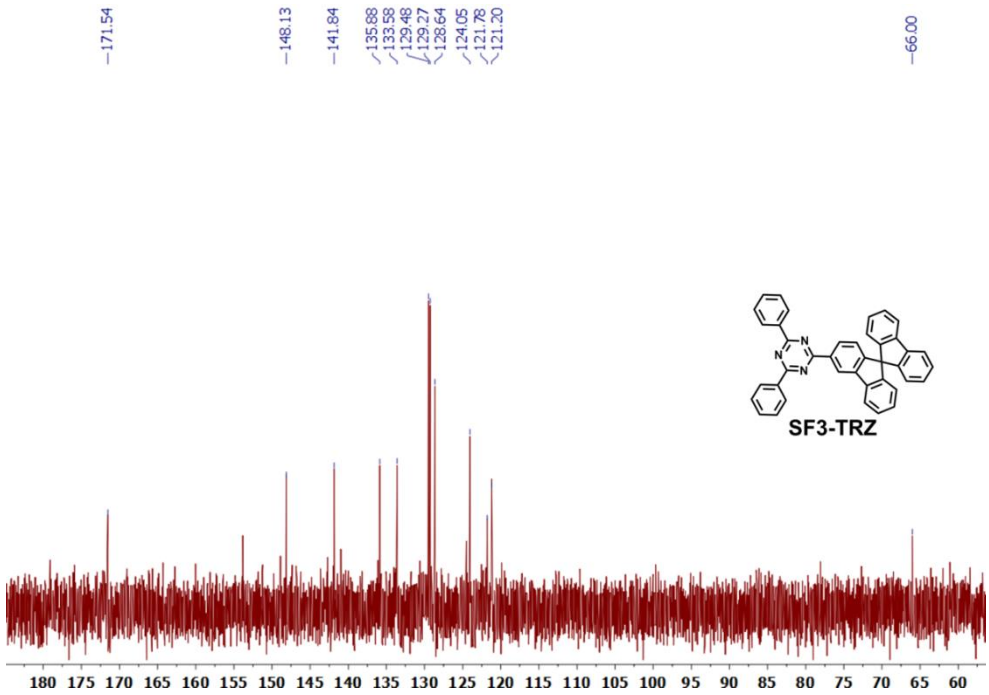

Supplementary Fig. 25 | ^{13}C NMR spectrum of SF3-TRZ.

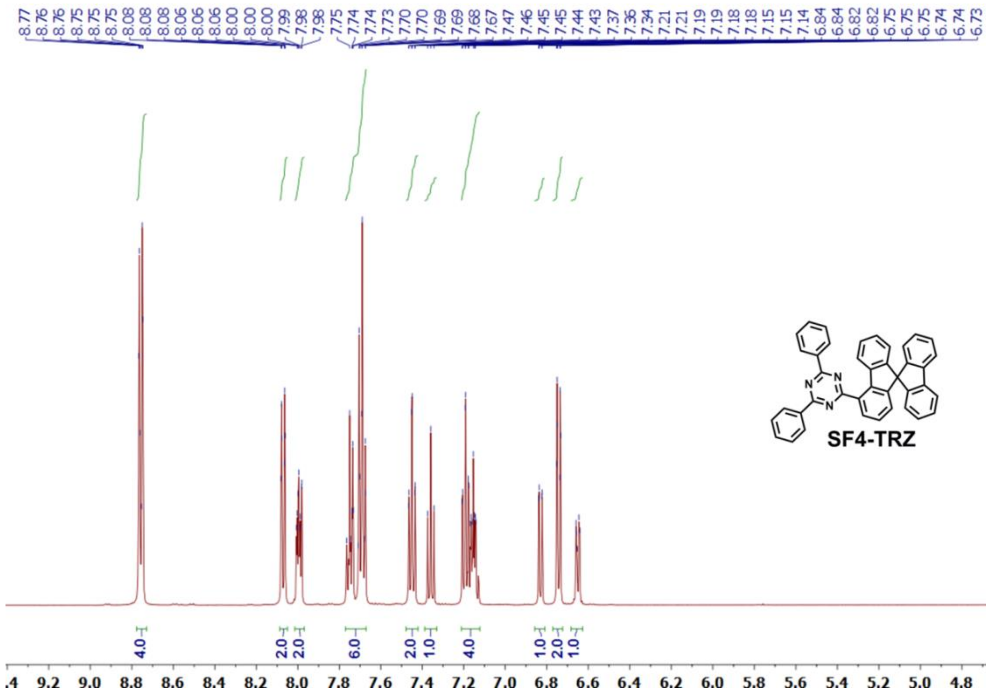

Supplementary Fig. 26 | ^{1}H NMR spectrum of SF4-TRZ.

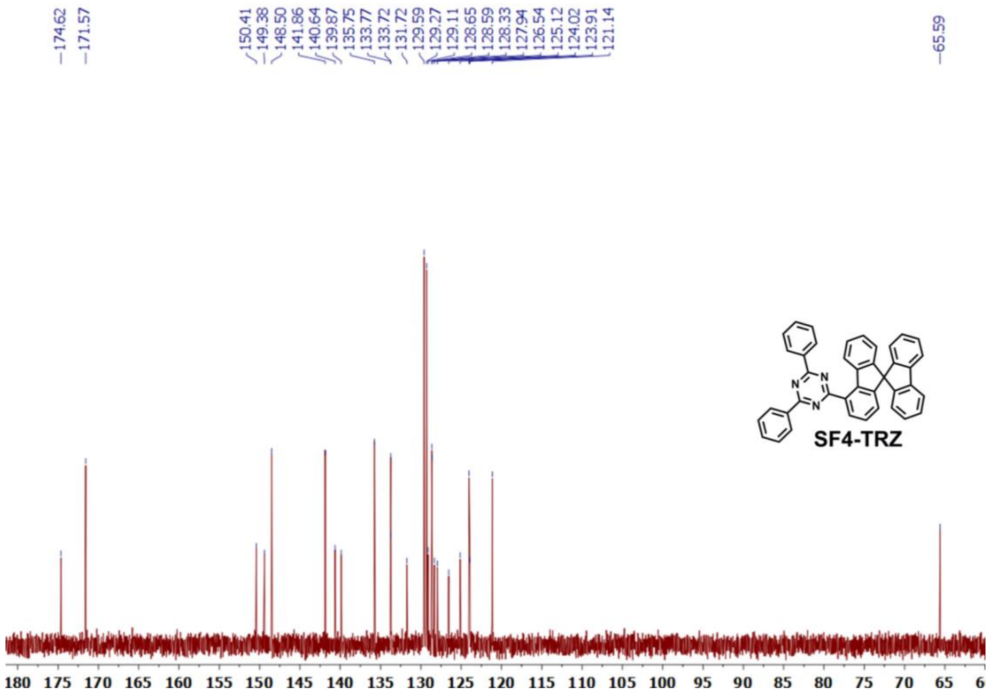

Supplementary Fig. 27 | ^{13}C NMR spectrum of SF4-TRZ.

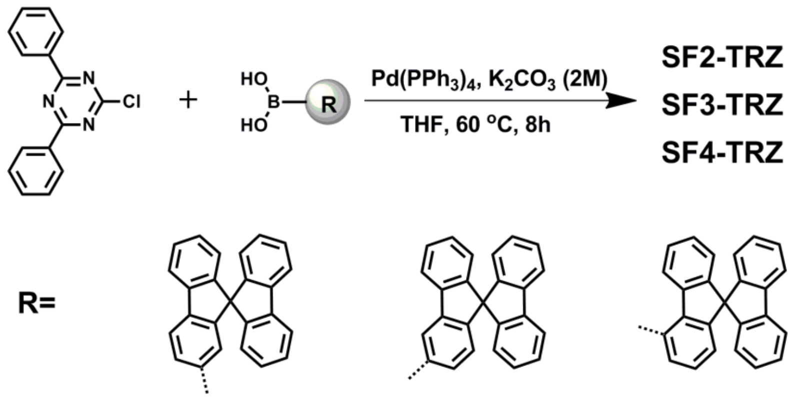

Supplementary Fig. 28 | Synthesis of SF2-TRZ, SF3-TRZ, and SF4-TRZ.

Supplementary Table 1 | Calculated HOMO, LUMO, bandgap, S_1, T_1 values.

Compound	HOMO (eV)	LUMO (eV)	Bandgap (eV)	$S_1@S_0$ (eV)	$T_1@S_0$ (eV)
**SF-TRZ2**	−5.70	−1.86	3.84	3.34	2.63
**SF-TRZ3**	−5.71	−1.79	3.92	3.45	2.82
**SF-TRZ4**	−5.67	−1.82	3.84	2.37	2.70

Supplementary Table 2 | Bond dissociation energies in the SF2-TRZ, SF3-TRZ, and SF4-TRZ.

Compound	Bond	Bond Dissociation Reaction	Average BDE (kcal/ mol)
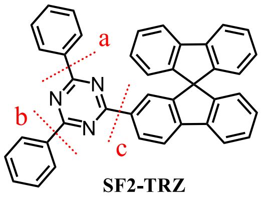 SF2-TRZ	a	$\text{SF2-TRZ}^{\cdot -} \rightarrow \text{SF2-TRZ}^{\cdot} + \text{Ph}^{-}$	115.5
		$\text{SF2-TRZ}^{\cdot -} \rightarrow \text{SF2-TRZ}^{-} + \text{Ph}^{\cdot}$	108.4
	b	$\text{SF2-TRZ}^{\cdot -} \rightarrow \text{SF2-TRZ}^{\cdot} + \text{Ph}^{-}$	115.8
		$\text{SF2-TRZ}^{\cdot -} \rightarrow \text{SF2-TRZ}^{\cdot} + \text{Ph}^{-}$	105.4
	c	$\text{SF2-TRZ}^{\cdot -} \rightarrow \text{SF}^{\cdot} + \text{TRZ}^{-}$	106.5
		$\text{SF2-TRZ}^{\cdot -} \rightarrow \text{SF}^{-} + \text{TRZ}^{\cdot}$	108.38
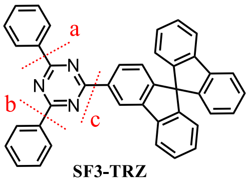 SF3-TRZ	a	$\text{SF3-TRZ}^{\cdot -} \rightarrow \text{SF3-TRZ}^{\cdot} + \text{Ph}^{-}$	113.5
		$\text{SF3-TRZ}^{\cdot -} \rightarrow \text{SF3-TRZ}^{-} + \text{Ph}^{\cdot}$	102.2
	b	$\text{SF3-TRZ}^{\cdot -} \rightarrow \text{SF3-TRZ}^{\cdot} + \text{Ph}^{-}$	113.5
		$\text{SF3-TRZ}^{\cdot -} \rightarrow \text{SF3-TRZ}^{\cdot} + \text{Ph}^{-}$	102.4
	c	$\text{SF3-TRZ}^{\cdot -} \rightarrow \text{SF}^{\cdot} + \text{TRZ}^{-}$	103.8
		$\text{SF3-TRZ}^{\cdot -} \rightarrow \text{SF}^{-} + \text{TRZ}^{\cdot}$	106.3
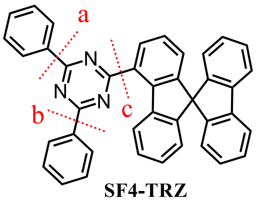 SF4-TRZ	a	$\text{SF4-TRZ}^{\cdot -} \rightarrow \text{SF4-TRZ}^{\cdot} + \text{Ph}^{-}$	114.8
		$\text{SF4-TRZ}^{\cdot -} \rightarrow \text{SF4-TRZ}^{-} + \text{Ph}^{\cdot}$	100.8
	b	$\text{SF4-TRZ}^{\cdot -} \rightarrow \text{SF4-TRZ}^{\cdot} + \text{Ph}^{-}$	114.8
		$\text{SF4-TRZ}^{\cdot -} \rightarrow \text{SF4-TRZ}^{\cdot} + \text{Ph}^{-}$	100.8
	c	$\text{SF4-TRZ}^{\cdot -} \rightarrow \text{SF}^{\cdot} + \text{TRZ}^{-}$	92.2
		$\text{SF4-TRZ}^{\cdot -} \rightarrow \text{SF}^{-} + \text{TRZ}^{\cdot}$	90.2

Supplementary Table 3 | HOMO and LUMO levels of reported TADF and other emitters.

Material	HOMO	LUMO
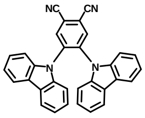 2CzPN	-5.80	-3.0
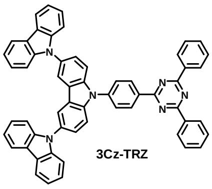 3Cz-TRZ	-5.90	-2.80
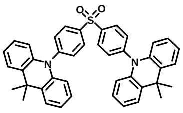 DMAC-DPS	-5.92	-2.92
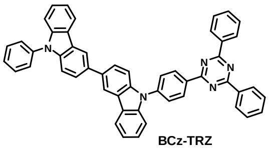 BCz-TRZ	-5.70	-2.70
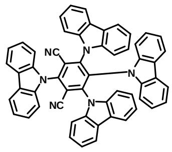 4CzIPN	-5.80	-3.40
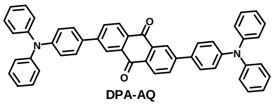 DPA-AQ	-5.90	-3.60
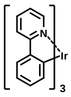 $Ir(ppy)_3$	-5.20	-2.70
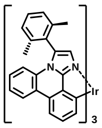 $Ir(dmp)_3$	-5.0	–
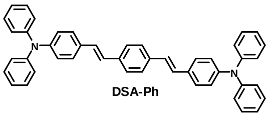 DSA-Ph	-5.40	-2.70

Supplementary Table 4 | Physical Properties of SF2-TRZ, SF3-TRZ and SF4-TRZ.

Compound	T_g/T_d[a)] (°C)	HOMO[b)] (eV)	LUMO[c)] (eV)	Bandgap[d)] (eV)	$S_1@S_0$[e)] (eV)	$T_1@S_0$[f)] (eV)
**SF-TRZ2**	131/398	−6.56	−3.27	3.29	3.18	2.53
**SF-TRZ3**	135/409	−6.54	−3.10	3.44	3.26	2.80
**SF-TRZ4**	132/384	−6.55	−3.23	3.32	3.04	2.65

[a)] T_g: glass transition temperatures; T_d: thermal decomposition temperatures. [b)] Measured by using AC-3 ultraviolet photoelectron spectrometer. [c)] LUMO levels were calculated from HOMO and E_g. [d)]Bandgap energy was estimated from the optical absorption edges of UV-Vis absorption spectra. [e)] Singlet energy was estimated from the onset peak of the fluorescence spectra. [f)] Triplet energy was estimated from the onset peak of the phosphorescence spectra.

Supplementary Table 5 | The PLQY of n-type hosts doped with TADF emitters.

Film (60 nm)	PLQY
**SF2-TRZ: 15 wt % 4CzIPN**	65%
**SF3-TRZ: 15 wt % 4CzIPN**	90%
**SF4-TRZ: 15 wt % 4CzIPN**	76%
**SF3-TRZ: 10 wt % DPA-AQ**	80%
**SF3-TRZ: 15 wt % BCz-TRZ**	82%
**SF3-TRZ: 30 wt % BCz-TRZ**	78%

Supplementary Table 6 | Zero-field mobility and Poole-Frenkel factor of neat films.

Material	μ_{0h} (10^{-9} cm^2 V^{-1} s^{-1})	μ_{0e} (10^{-5} cm^2 V^{-1} s^{-1})	β_h (10^{-4} cm$^{0.5}$ V$^{-0.5}$)	β_e (10^{-3} cm$^{0.5}$ V$^{-0.5}$)
**SF2-TRZ**	7.50	6.68	2.70	1.74
**SF4-TRZ**	11.9	2.11	5.14	0.4

Supplementary Table 7 | Zero-field mobility and Poole-Frenkel factor of neat and doped films.

Material	μ_{0h} (10^{-9} cm^2 V^{-1} s^{-1})	μ_{0e} (10^{-9} cm^2 V^{-1} s^{-1})	β_h (10^{-4} cm$^{0.5}$ V$^{-0.5}$)	β_e (10^{-3} cm$^{0.5}$ V$^{-0.5}$)
**SF3-TRZ**	8.81	6990	5.56	1.42
***m*CBP**	305	—	11.22	—
**SF3-TRZ: 15 wt% 4CzIPN**	35.1	25.4	26.99	0.44
***m*CBP: 15 wt% 4CzIPN**	10.3	0.0101	87.88	0.41

Supplementary Note 1 | Synthesis of SF2-TRZ, SF3-TRZ, and SF4-TRZ

Synthesis of 2-(9,9'-spirobi[fluoren]-2-yl)-4,6-diphenyl-1,3,5-triazine (SF2-TRZ). A mixture of 2-chloro-4,6-diphenyl-1,3,5-triazine (1.34 g, 5.01 mmol), 9,9'-spirobi[fluoren]-2-ylboronic acid (1.89 g, 5.26 mmol), $Pd(PPh_3)_4$ (0.3 g, 0.27 mmol) and potassium carbonate (1.38 g, 10.02 mmol) in 30 mL of THF and 10 mL of distilled water in a 100 mL round bottom was refluxed for 6 hours under argon. The mixture was extracted with chloroform. The combined organic extracts were dried over Na_2SO_4 and concentrated by rotary evaporation. The crude product was purified by column chromatography on silica gel using 1: 4 ethyl acetate/petroleum as eluent to afford a white solid SF2-TRZ (2.36 g, 86 % yield). ^{1}H NMR (500 MHz, DMSO-d_6) δ (ppm): 8.89 (d, *J* = 8.0 Hz, 1H) 8.55 (d, *J* = 8.5 Hz, 4H) 8.34 (d, *J* = 8.0 Hz, 1H) 8.20 (d, *J* = 7.5 Hz, 1H) 8.13 (d, *J* = 7.5 Hz, 2H) 7.86 (s, 1H) 7.70-7.57 (m, 6H) 7.52-7.42 (m, 3H) 7.26-7.14 (m, 3H) 6.73 (d, *J* = 7.5 Hz, 2H) 6.65 (d, *J* = 7.5 Hz, 1H). ^{13}C NMR (125 MHz, DMSO-d_6) δ (ppm): 171.3, 171.2, 150.0, 149.1, 148.0, 146.7, 141.8, 140.5, 135.7, 135.5, 133.4, 129.9, 129.7, 129.4, 128.9, 128.7, 124.0, 123.5, 122.1, 121.6, 121.3, 66.01. HRMS *m/z*: 547.26 $[M]^+$. Anal. calcd for $C_{40}H_{25}N_3$ (%): C 87.73, H 4.60, N 7.67; found: C 87.70, H 4.62, N 7.69.

Synthesis of 2-(9,9'-spirobi[fluoren]-3-yl)-4,6-diphenyl-1,3,5-triazine (SF3-TRZ). SF3-TRZ was synthesized according to the same procedure followed for SF2-TRZ using 9,9'-spirobi[fluoren]-3-ylboronic acid (1.89 g, 5.26 mmol) instead of 9,9'-spirobi[fluoren]-2-ylboronic acid. After evaporation of the solvent, the crude product was subjected to column chromatography on silica gel using 1:4 dichloromethane/petroleum as eluent to afford a yellow solid with a yield of 82%. ^{1}H NMR (500 MHz, DMSO-d_6) δ (ppm): 9.31 (s, 1H) 8.81 (d, *J* = 8.0 Hz, 4H) 8.54 (d, *J* = 8.0 Hz, 1H) 8.38 (d, *J* = 7.5 Hz, 1H) 8.09 (d, *J* = 8.0 Hz, 2H) 7.80-7.65 (m, 6H) 7.54-7.41 (m, 3H) 7.27-7.11 (m, 3H) 6.74-6.65 (m, 3H). ^{13}C NMR (125 MHz, DMSO-d_6) δ (ppm): 171.5, 148.1, 141.8, 135.8, 133.5,

129.5, 129.2, 128.6, 124.0, 123.5, 122.7, 121.2, 66.0. HRMS *m/z*: 547.26 $[M]^+$. Anal. calcd for $C_{40}H_{25}N_3$ (%): C 87.73, H 4.60, N 7.67; found: C 87.71, H 4.62, N 7.65.

Synthesis of 2-(9,9'-spirobi[fluoren]-4-yl)-4,6-diphenyl-1,3,5-triazine (SF4-TRZ). SF4-TRZ was synthesized according to the same procedure followed for SF2-TRZ using 9,9'-spirobi[fluoren]-4-ylboronic acid (1.89 g, 5.26 mmol) instead of 9,9'-spirobi[fluoren]-2-ylboronic acid. After evaporation of the solvent, the crude product was subjected to column chromatography on silica gel using 1:4 dichloromethane/petroleum as eluent to afford a yellow solid with a yield of 75%. ^{1}H NMR (500 MHz, DMSO-d_6) δ (ppm): 8.76 (d, *J* = 7.0 Hz, 4H) 8.08 (d, *J* = 7.5 Hz, 2H) 8.01-7.97 (m, 2H) 7.77-7.67 (m, 6H) 7.48-7.42 (m, 2H) 7.38-7.33 (m, 1H) 7.21-7.12 (m, 4H) 6.84 (d, *J* = 7.5 Hz, 1H) 6.75 (d, *J* = 7.5 Hz, 2H) 6.66 (d, *J* = 7.5 Hz, 1H). ^{13}C NMR (125 MHz, DMSO-d_6) δ (ppm): 174.6, 171.5, 150.4, 149.3, 148.5, 141.8, 140.6, 139.8, 135.7, 133.5, 131.7, 129.5, 129.2, 129.1, 128.7, 128.6, 127.9, 126.5, 125.1, 124.0, 123.9, 121.1, 65.59. HRMS *m/z*: 547.22 $[M]^+$. Anal. calcd for $C_{40}H_{25}N_3$ (%): C 87.73, H 4.60, N 7.67; found: C 87.70, H 4.61, N 7.66.

Supplementary Note 2 | Charge carrier transport properties

The *J–V* characteristics show two distinct regions at low and high biases, such as Schottky thermionic region and SCLC region, respectively. As voltage increases, the *J–V* characteristics switch to the SCLC and the SCLC can be expressed as

$$J = \frac{9}{8}\varepsilon\varepsilon_0\mu\frac{V^2}{L^3}\ , \qquad (1)$$

where V is the applied voltage, ε and ε_0 are the relative dielectric constant and the permittivity of the free space, respectively, and L is the thickness of the organic layer. The carrier mobility is affected by the energetic disorder due to the interaction of each hopping charge with randomly oriented and randomly located dipoles in the organic thin film. Therefore, the mobility is dependent on the electric field and can be expressed by a Poole-Frenkel PF equation,

$$\mu = \mu_0 \exp\left(\beta\sqrt{\frac{V}{L}}\right), \qquad (2)$$

where μ_0 is the zero-field mobility and β is Poole-Frenkel factor. From the combination of equations (1) and (2), the field dependent SCLC can be easily expressed by

$$J = \frac{9}{8}\varepsilon\varepsilon_0\mu_0 \exp\left(\beta\sqrt{\frac{V}{L}}\right)\frac{V^2}{L^3}. \quad (3)$$

The detailed data are summarized in **Supplementary Tables 6** and **7.**

Supplementary Note 3 | Optical simulations

Optical simulations are performed using SETFOS 4.5 software in order to extract the recombination zone profile and position and to calculate the light outcoupling as well as the optical channel losses in the SF3-TRZ (n-type host) and *m*CBP (p-type host)-based OLEDs. The profile and the position of the recombination zone in the devices were extracted by fitting the measured EL spectra to an optical model. In this optical model the excitons are modeled as isotropic radiative dipoles driven by the multiple reflections inside the devices. The power radiated from a dipole at a certain wavelength is weighed by the photoluminescence spectrum of the emitting layer. Using the same approach cited above, the dissipated power as a function of the wavelength and the in-plane wave vector can be calculated. The contribution of the modes is obtained by integrating the dissipated power.

The simulated EL spectra for *m*CBP and SF3-based OLEDs presented in Supplementary Figs. 21a and 21c, respectively, show a good agreement with the experimental EL spectra. The extracted recombination zone position and shape for *m*CBP and SF3-based OLEDs are shown in Supplementary Figs. 21b and 21d, respectively. When a p-type host material is used, the recombination zone is situated at the cathode side (Supplementary Fig. 21b). Interestingly, when the n-type host material is used, the recombination zone is shifted to the other side away from the cathode due to the faster electron mobility (Supplementary Fig. 21d). In addition, the profile of the recombination zone is wider in the case of the n-type-based OLED due to a good charge carrier balance in this device.

The shift of the recombination zone affects the light trapping and outcoupling of the devices. In order to quantify the light trapping and outcoupling, the dissipated power as a function of the wavelength and the in-plane wave vector is calculated and shown in Supplementary Figs. 21e and 21f

for *m*CBP and SF3-based OLEDs, respectively. The different optical loss channels are clearly identified for both devices. The percentage of the optical power coupled to the different optical channels in *m*CBP-based OLED and SF3-based OLED are shown in Supplementary Figs. 21g and 21h, respectively. In the *m*CBP-based OLED, the Surface Plasmon Polariton (SPP) loss is dominant (Supplementary Fig. 21e) and reach 69% whereas in the SF3-based OLED, due to the shift of the recombination zone away from the cathode, the SPP loss is decreased to 12% and the waveguide mode becomes dominant (Supplementary Fig. 21f). In addition, the substrate mode and the external light outcoupling is enhanced. The outcoupled light in the SF3-based OLED is found to be 19% against 9% for the *m*CBP-based OLED which is consistent with the experimental values.

Supplementary References

1. Hirata, S. *et al.* Highly efficient blue electroluminescence based on thermally activated delayed fluorescence. *Nat. Mater*. **14,** 330-336 (2015).
2. Cui, L. -S. et al. Pure Hydrocarbon Hosts for ≈100% Exciton Harvesting in Both Phosphorescent and Fluorescent Light-Emitting Devices. *Adv. Mater*. **27,** 4213-4217(2015).
3. Zhang, Q. *et al.* Efficient blue organic light-emitting diodes employing thermally activated delayed fluorescence. *Nat. Photonics*, **8,** 326-332 (2014).
4. Uoyama, H. *et al.* Highly efficient organic light-emitting diodes from delayed fluorescence. *Nature* **492,** 234-238 (2012).
5. Zhang, Y., Lee, J. & Forrest. S. R. Tenfold increase in the lifetime of blue phosphorescent organic light-emitting diodes. *Nat. Commun.* **5,** 5008 (2014).
